# Comparing Sound-Field Speech-Auditory Brainstem Response Components between Cochlear Implant Users with Different Speech Recognition in Noise Scores

**DOI:** 10.22037/ijcn.v16i2.27210

**Published:** 2022-03-14

**Authors:** Farnoush JAROLLAHI, Ayub VALADBEIGI, Bahram JALAEI, Mohammad MAAREFVAND, Masoud MOTASADDI ZARANDY, Hamid HAGHANI, Zahra SHIRZHIYZN

**Affiliations:** 1 Department of Audiology, School of Rehabilitation Sciences, Iran University of Medical Sciences, Tehran, Iran; 2 Cochlear Implant Center and Department of Otorhinolaryngology, Amir Aalam Hospital, Tehran University of Medical Sciences, Tehran, Iran; 3 Department of Biostatistics, School of Public Health, Iran University of Medical Sciences, Tehran, Iran.; 4 Department of Medical Physics and Biomedical Engineering, School of Medicine, Tehran University of Medical Sciences, Tehran, Iran

**Keywords:** Cochlear Implant, Auditory Brainstem Response, Speech Perception, Noise

## Abstract

**Objectives:**

Many studies have suggested that cochlear implant (CI) users vary in terms of speech recognition in noise. Studies in this field attribute this variety partly to subcortical auditory processing. Studying speech-Auditory Brainstem Response (speech-ABR) provides good information about speech processing; thus, this work was designed to compare speech-ABR components between two groups of CI users with good and poor speech recognition in noise scores.

**Materials & Methods:**

The present study was conducted on two groups of CI users aged 8-10 years old. The first group (CI-good) consisted of 15 children with prelingual CI who had good speech recognition in noise performance. The second group (CI-poor) was matched with the first group, but they had poor speech recognition in noise performance. The speech-ABR test in a sound-field presentation was performed for all the participants.

**Results:**

The speech-ABR response showed more delay in C, D, E, F, O latencies in CI-poor than CI-good users (P <0.05), meanwhile no significant difference was observed in initial wave (V(t= -0.293, p= 0.771 and A (t= -1.051, p= 0.307). Analysis in spectral-domain showed a weaker representation of fundamental frequency as well as the first formant and high-frequency component of speech stimuli in the CI users with poor auditory performance.

**Conclusions:**

Results revealed that CI users who showed poor auditory performance in noise performance had deficits in encoding the periodic portion of speech signals at the brainstem level. Also, this study could be as physiological evidence for poorer pitch processing in CI users with poor speech recognition in noise performance.

## Introduction

Everybody in real-life routinely encounters background noise, and increasing noise levels in the industrialization of today's society have led to interfering in communication and learning. Speech recognition in noise conditions is among the conditions causing problems for both children with hearing disorders and normal-hearing (NH) children. Cochlear implant (CI) is a device used for direct stimulation of auditory nerve for hearing restoration in severe to profound hearing loss, but a large number of studies on this field have suggested that CI users vary in speech recognition, as some of them understand speech very well while some others are very poor after implantation. Generally, most CI users have good speech recognition in quiet environments, but this ability is significantly compromised in the presence of background noise (1, 2). There are many factors that may cause variation in speech recognition of CI users, including degree and duration of hearing loss, the age of cochlear implantation, cochlear prosthesis, auditory nerve, brainstem, or reorganization of the higher central auditory pathway. The outcome of CI is determined by recognizing monosyllabic words or sentences in quiet and noise conditions. However, behavioral performance shows the combination of sensory and cognitive processes; thus, in the case of CI users, it is not well understood how variation in speech recognition is related to different specific levels of auditory processing. Considering speech recognition problems in CI users as well as unique features of this complex sound relative to simpler sounds, such as clicks and tone burst, understanding of how this particular sound is processed in different levels of the central auditory pathway, will likely contribute to our intuition regarding speech recognition problem (2-4). 

Speech sounds are a stream of acoustical elements produced at a rate of three to six syllables per second. Complicated processing is needed to encode these elements and translate them as meaningful words in the cortex. Neural bases of speech perception are primarily located in the cerebral cortex. However, before these sounds are registered and stored in long-term memory, relevant acoustical elements of them must be represented as neural messages encoded through subcortical structure and delivered to the auditory cortex. Regarding the major role of brainstem processing in speech stimuli on the one hand and insufficient information about speech sound processing, especially at the brainstem level in CI users, on the other hand, this study was conducted to investigate how sound stimuli are processed at the brainstem level (5).

Event-related potentials (ERPs) can provide enough information about the neural processing of stimuli at different levels of the auditory pathway (4). Many studies that recorded scalp-evoked response to speech sound have suggested that auditory brainstem response shows important features and basic acoustic elements of speech sound. Speech-auditory brainstem response (speech-ABR) as a promising, objective, and noninvasive audiological technique is used for measuring temporal and spectral encoding of a speech sound at brainstem level (6). Speech-ABR is a highly replicable method for the assessment of speech sound processing, and this response is mature by school-age children at five years old (7). Despite the existence of many complex sounds, /da/ syllabic sound is the most common and well-known speech sound used in more studies. Brainstem response to speech sound can be used as an index for neural synchronization in an individual with neural impairments. Speech-ABR is known to be language-, music-, experience-, and cognitive-dependent (8). 

Takwa Gabr et al. performed speech-ABR on two groups of CI users with good and poor cortical evoked potentials fitted with unilateral CIs, and they reported that speech-ABR provided a clinical tool showing the role of the brainstem in speech stimuli, contributing to cortical processing (1). Also, other studies have shown a relationship between speech in noise ability and auditory brainstem responses to speech stimuli. These studies have shown that subcortical neural encoding of the speech signal is a key factor for the determination of speech in noise ability (9). A previous study reported that speech-ABR could be used as neural synchrony in impaired subjects, such as individuals with learning impairment, hearing loss, and children with reading problems (10). Among different processing of brainstem structure, phase-locked activity to F0 and formant transition portion in speech-ABR test contribute to the determination of speech recognition in noise ability (11). The capability of the speech-ABR test for measuring neural synchrony and the relationship between speech recognition in noise and processing of sound stimuli in the brainstem motivated us to suppose that speech-ABR could provide a biological marker for CI users with different speech recognition in noise performance. Thus, it was assumed that poor speech recognition in noise performance results in part from impaired neural encoding, and accordingly, it is expected to observe a correlation of degraded brainstem neural encoding in CI users with different recognition in noise performance (9). Therefore, this study was designed to compare sound-field speech-ABR components between two groups of CI users with different speech recognition in noise performance to test the hypothesis that CI user with poor speech recognition in noise performance has specific dysfunction at the brainstem level.

## Materials & Methods

**Subjects**

In the current study, 30 unilateral CI users aged 8-10 years old were recruited and assigned to two groups. The first group (**CI-good**) consisted of 15 prelingual children with a mean age of 8.83 (± 0.63) years. The second group (**CI-poor**) consisted of 15 children with a mean age of 9.08 (± 0.67) years who were matched in terms of chronological age, age at cochlear implantation, duration of CI usage, CI prosthesis type, and speech recognition in quiet with the first group but they had poor speech recognition in noise performance. All the participants were chosen from the ×× Cochlear Implant Center in ×, ×. Consents were taken from all parents of children before administration of the tests, and to ensure ethical considerations related to the treatment, this research was approved by the Ethics Committee of × University of Medical Sciences (×), in accordance with the 1975 Declaration of Helsinki and its later amendments. This study was conducted over a period of five months from January to May 2018 at the School of Rehabilitation Sciences, × University of Medical Sciences. All the children had a prelingual onset of bilateral profound sensory-neural hearing loss and received unilateral cochlear implantation in the right ear. In this study, only the validated Persian version of the speech in noise test was used for children, called as Persian Auditory Recognition of Word in Noise (PARWIN) test. They were then divided into two subgroups of CI-good and CI-weak according to the results of the PARWIN test. Based on the results of PARWIN test, those obtained scores lower than two standard deviations of the average of the CI-good group were included in the CI-poor group.

Inclusion criteria for the first group (**CI-Good**): This group included CI users with bilateral profound congenital sensory-neural hearing loss before implantation who were not successfully treated with a hearing aid for at least six months. These participants used a nucleus prosthesis (CI24RE) and advanced combination encoder (ACE) processing strategy with an omnidirectional microphone in the right ear for at least three years. All children were -monolingual, right-handed, and had no history of head trauma, cognitive problems, neurologic impairment, growth-related diseases, and psychological disorders. The second group (**CI-poor**) was matched with the first group, but they had poor speech recognition of words in noise according to the results of the PARWIN test. Children who were unwilling to cooperate and perform the tests, as well as those with general health problems and conductive disorders, were excluded from the study.

Mean and standard deviation of the chronological age of two groups of CI users, age at the time of implantation, duration of CI usage, and the age of identification of hearing loss are presented in Table 1. 

**Table 1:** The mean and standard deviation of chronological age, age at the time of CI, duration of CI usage, and identification of HL in two study groups

**CI- Poor**	**CI- Good**	**Descriptive Statistics**
(Mean ±SD, y)	(Mean ±SD, y)	
9.08 ± 0.67	8.83± 0.63	Chronological age
4.48 ± 0.75	4.25± 0.78	Age at cochlear implantation
4.53 ± 0.64	4.61 ± 0.79	Duration of CI usage
1.15 ± 0.29	1.33 ± 0.37	Identification of HL

CI: Cochlear Implant, HL: Hearing Loss, y: year

**Materials & Methods**

For all the participants, one month before the administration of the tests, CI electrode impedance, and neural response telemetry were measured and verified so that they could use speech processors perfectly in experiment sessions. The experiments were performed in two parts: behavioral and electrophysiological assessments. Before administrating the test, devices and loudspeaker output were calibrated by the sound level meter (B&K model 2209). Also, all tests were done in an anechoic booth to decrease background noise and diffusion of sound. Depending on participants' heads and level of ears, the loudspeaker's position was set at a 45-degree angle and one meter away from them. 

At the beginning of the study, behavioral tests were performed involving sound-field audiometry with a warble tone and Word Discrimination Score (WDS) in quiet using a clinical audiometer (Interacoustic AC40) Pejvak Ava loudspeaker. The WDS test was administered at each participant's most comfortable level (MCL). A list of 25 monosyllabic Persian words was played at MCL via an MP3 player connected to audiometers. Then, the participants were asked to repeat presented words (12). Then, the PARWIN test was performed for all participants. This test is designed to assess word recognition in noise among Persian children of 6 - 12 years of age. Test results were recorded on a CD and presented via an audio player connected to an audiometer, while the level of output intensity and test ear were adjusted. This test consisted of 35 monosyllabic words in the presence of a six-speaker babble noise, in which the signal-to-noise ratio decreased from + 24 to +0 dB in 4-dB steps. At each Signal-to-Noise Ratio (SNR) level, five monosyllabic words were presented to the participants, and they were asked to repeat those words. The test measured SNR for 50% of word recognition. 

Needed SNR to obtain a level of 50% of correct recognition regarding Persian monosyllabic words in background noise was determined by the Spearman-Karber Equation: SNR (50) = I + 1/2(d) - d (#correct)/w.

 Where, I is initial SNR intensity (24dB), d is intensity step (4dB), correct represents correctly repeated words, and w is the number of words in each step (5word). According to this formula, in the PARWIN test, there was a table related to calculated values of SNR for all 35 words. Hence, to measure SNR for each participant, it is only required to count the total number of correctly repeated words. This test consists of three lists: list one is for the right ear, list two is for the left ear, and list three is for a binaural condition, and all should be administered at MCL (13).

**Figure1:**
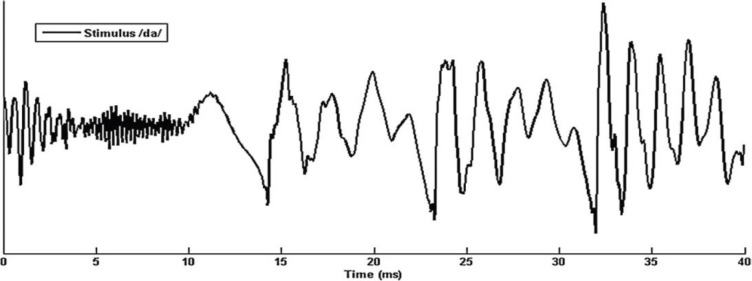
Time-domain of synthesized stop consonant /da/ with 40 ms

After administrating behavioral tests, an electrophysiological test (speech-ABR) was performed for all subjects. A Biologic Navigator Pro (Natus Medical Inc., San Carlos, CA, USA) device was used to measure speech-ABR test. Each participant was instructed at the beginning of the test, and they were asked to sit quietly on a chair and do not talk or move. To achieve decreased physical movements and more relaxation, a mute animation movie was displayed for them on the screen placed in front of them. Ag-Excl electrodes were located on the skull for recording auditory evoked potentials. To decrease electrodes' impedance, the place of electrodes was cleaned using skin cleanser gel. For a better recording of evoked potentials, the impedance of the electrodes was kept less than 5 kΩ during the test, and the inter-electrode difference was set below than 3 kΩ. The intensity of the stimuli was presented at 50 dB sensation level (SL) through a loudspeaker at the alternating polarity and a rate of 9.1 per second. Other characteristics of evoked potentials parameters included epoch time of 85.33 ms, 15 ms pre-stimulus, and online filter setting with 100-2000 Hz. For each participant, a total of 4000 sweeps with artifact-free responses were averaged. For controlling of artifact from CI prosthesis with respect to a pilot study, the electrode array, including Vertex (CZ) as non-inverting, earlobes in contralateral as inverting, or references and forehead (Fpz) as a ground electrode was used. Synthesized stop consonant /da/ with 40 ms duration was used as speech stimuli in this study (Figure 1).

/da/ synthetic sound has three parts, including initial noise burst, formant transition, and steady-state vowel. Steady-state vowel contains F0 with 103 to 125 Hz rising linearly. In addition to F0, these speech stimuli have high formants, such as F1 is rising linearly from 220 to 720 Hz and HF containing higher frequency from 1700 to 4500 Hz. These responses represent a neural event of sound synchronously, which is phase-locked to the acoustical feature of speech stimuli. 

The speech-ABR wave extracted from /da/ synthetic speech has seven peaks: wave V and A evoked from the initial part of stimuli onset or the start of the sound, peak C as an indicator of the transient portion from consonant to a vowel part, D, E, and F peaks evoked by the sustained portion of stimuli and also frequency following response (FFR), and finally, O peak showing response to ending of stimuli. Initials of response show synchrony of neural response to stimulus and sustain portion of response indicators of cumulative phase-locking activities of brainstem coinciding with the period of a speech stimulus (14). The speech-ABR parameters, including latency, the amplitude of all the waves, and also slope and area of V/A complex, were measured for more accurate analysis. Furthermore, responses at the spectral domain, including the FFR component in F0, F1, and HF, were measured. 

The data obtained from the speech-ABR test were processed using MATLAB software version 2010 (Math Works, Inc., Natick, Massachusetts, USA). For statistical analysis, IBM SPSS software version 25 (SPSS Inc., Chicago, USA) was used at a significance level of 0.05. In the two groups of CI users, the mean and standard deviation of the speech-ABR test were calculated. To compare the variables with normal distribution, the independent samples t-test was utilized, while for variables with the abnormal distribution of data, the Kruskal-Wallis test was applied.

## Results

**Behavioral Measures**

The mean of word discrimination scores in quiet was equal to 72.93% ±6.88 and 68.93% ±5.89 for CI-good and CI-poor groups, respectively. There was no significant difference in word discrimination scores in the quiet between the two groups of CI users. Descriptive statistics, including mean and SD for hearing level threshold at 500, 1k, 2k, and 4 kHz, WDS, and PARWIN test for two groups of CI users are outlined in Table 1. 

**Table 1:** condition. Mean and SD of the hearing threshold level, WDS, and PARWIN test

**CI- Poor**	**CI- Good**	**Descriptive Statistics**
(Mean ±SD, y)	(Mean ±SD, y)	
32.66 ± 8.63	28.66 ± 6.11	500 Hz (threshold)
22.00 ± 9.02	22.33±6.51	1000 Hz (threshold)
23.00 ± 7.97	20.33±7.18	2000 Hz (threshold)
26.66 ± 6.72	21.00±7.83	4000 Hz (threshold)
68.93 ± 5.89	72.93±6.88	WDS (percent)
12.93 ± 2.52	6.91±2.23	PARWIN (SNR)

CI: Cochlear Implant, WDS: Word Discrimination Score, PARWIN: Persian Auditory Recognition Word in Noise

**Figure2:**
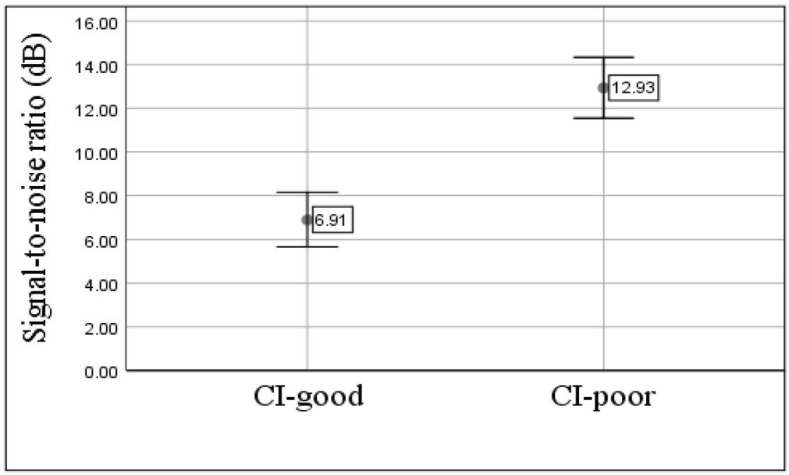
Mean and standard deviation of Persian Auditory Recognition Word in Noise (PARWIN) test in two groups of cochlear implant (CI) users in this study

Comparison of the results of the PARWIN test scores in the two groups revealed that CI-good group achieved lower SNR ratios or better auditory performance in noise for discrimination of words in noise than CI-poor group. These results showed a significant difference between the two groups of CI users after adding the noise (P <0.05) (Figure 2). 

**Sound-Field Speech-ABR** **Measures**

**Figure3:**
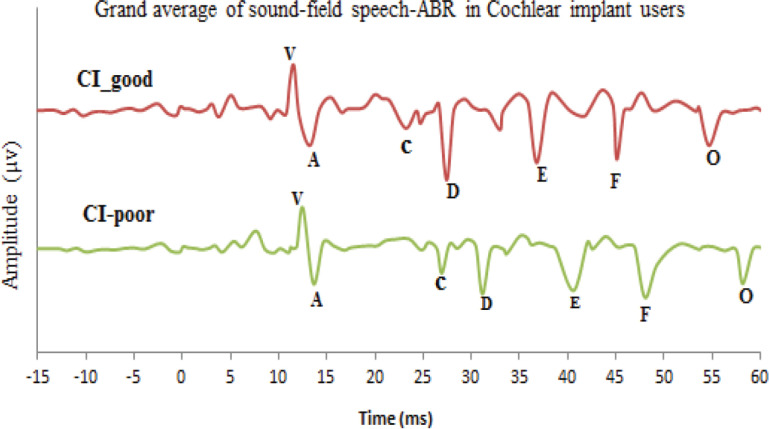
Grand average of speech- Auditory Brainstem Response (ABR) test in two groups of cochlear implant (CI) users in this study

Results of independent samples t*-*test revealed that the CI-poor group had longer absolute latency for sustained and offset peaks of the speech-ABR test compared to CI-good users (p<0.05). Analysis of transient portion of response showed longer latency of V and A peaks in CI-poor group, but these differences were not statically significant between the two groups. The t- and p-values were obtained as -0.293, p= 0.771 and t= -1.051, p= 0.307, respectively for V and A peaks between the two groups. For deeper assessment, duration, amplitude, slope, and area of V/A complex were measured as indices of onset responses. Independent samples t-test indicated no significant differences in A/V, duration, and slope between the two groups of CI users. The mean, SD, and p-value of all peaks of speech-ABR are shown in Table 2. The grand average of sound-field speech-ABR waveforms for children in CI-good and CI-poor groups are shown in Figure 3.

The results showed that the amplitude of the FFR portion, including D, E, and F waves, was significantly lower in the CI-poor group than CI-good group; however, analyses of other waves showed that there was no significant difference in initial waves (V and A), transition part (C), and offset of response. Mean, SD, and p-value for the amplitude of waves in the two groups are shown in Table 2. To measure the response of formant transition of speech stimuli, spectral amplitude of fundamental frequency (80-121 Hz) of formant transition and its harmonics, including F1 (ranged from 454 to719 Hz), and high frequency (HF) (ranged from 721 to1155 Hz), the Fourier analysis was performed. In the group of CI users with different recognition in noise performance, it was found that spectral encoding of F0 speech stimuli (t= 3.602, p= 0.001), F1 range (t= 3.027, p= 0.005), and HF response components (t=2.493, p=0.019) were affected (Table 2).

**Table2:** Mean, SD, and P-value for latency, amplitude, V/A complex, and spectral measures

	**CI-Good**		**CI-Poor**		
	Mean	SD	Mean	SD	p
**Latency(ms)**					
V	11.96	0.49	12.02	0.52	0.771
A	13.43	0.32	13.93	0.75	0.307
C	24.36	0.41	27.17	0.68	0.000
D	27.79	0.58	31.48	0.51	0.000
E	36.59	0.87	40.19	0.33	0.000
F	45.65	1.03	48.70	0.59	0.000
O	54.16	0.48	58.06	0.28	0.000
**Amplitude(** **µ** **v)**					
V	0.12	0.04	0.11	0.02	0.561
A	-0.14	0.05	-0.12	0.02	0.456
C	-0.09	0.03	-0.08	0.03	0.515
D	-0.26	0.07	-0.21	0.04	0.020
E	-0.24	0.04	-0.20	0.04	0.024
F	-0.18	0.05	-0.14	0.03	0.012
O	-0.12	0.03	-0.11	0.02	0.482
**V/A Complex measure**					
Duration(ms)	1.47	0.28	1.65	0.95	0.472
Amplitude(µv)	0.26	0.06	0.24	0.03	0.313
Slope (µv/ms)	-0.18	0.06	-0.12	0.15	0.138
Area (µv×ms)	0.19	0.06	0.21	0.09	0.558
**Spectral magnitudes (** **µ** **v)**					
F0	7.04	2.44	4.62	0.89	0.001
F1	2.25	1.01	1.34	0.57	0.006
HF	0.44	0.24	0.25	0.17	0.019

## Discussion

In the current study, brainstem function in processing auditory with /da/ speech stimuli was investigated in CI users. This study focused on comparing sound-field speech-ABR components between the two groups of CI users who had different speech recognition in noise ability. The main finding of the current study revealed a difference in neural encoding of sustained FFR and offset part of speech stimuli in the speech-ABR test. This discrepancy was not seen in the transient peak of speech-ABR. These results were in line with the study by Takwa Gabr et al. who reported that in two groups of CI users with different cortical responses, the sustained portion of speech-ABR was different between the two groups, but there was no significant difference in the transient portion (1). 

CI users are faced with a deficiency in processing short-duration stimuli like a transient portion of speech sound; thus, in this study, no differences were found between the two groups of CI users. The results of this study were in agreement with the study by Xin Luo who showed that temporal integration for the short duration was limited than long stimulus in CI users (15). 

As mentioned earlier, FFR reflects the phase of locking of brainstem activity to stimuli periodicity, and differences between the two groups of CI users in this study showed that in the CI-poor group, brainstem response to speech sound was not phase-locked or at least was weaker than the CI-good group. Many studies focusing on FFR recording in humans reported that the brainstem had an essential role in the processing of speech and speech-like sounds. Therefore, one of the reasons that may have had a role in different capabilities of CI users is different FFR processing at brainstem level to speech stimuli (16, 17). Several factors can be mentioned for justification of these results. Previous studies showed that subcortical auditory processing has a plastic and adaptable nature influenced by learning and cognitive processes; thus, it probably has a top-down effect, such as limited language experience, and phonological awareness can influence brainstem processing in CI users. On the other hand, deficient processing of initials at the brainstem level is likely to make abnormal inputs for central structure (bottom-up processing). Finally, it can be concluded that timing deficits observed in CI users with poor brainstem decoding likely result from a combination of both bottom-up and top-down regulated processes (18). 

Other onset responses, including characteristics of V and A peaks (VA complex), such as duration, amplitude, slope, and area, were also analyzed. The findings showed no significant difference between the two groups, supporting previously mentioned causes regarding the processing of short-duration stimulus in CI users. The results of the amplitude of speech-ABR test showed that the amplitude of FFR waves (D, E, and F) was significantly lower in the CI-poor group than the CI-good group; however, the amplitude of the formant transition portion did not differ significantly. These results were in line with the study by Takwa Gabr et al., who reported that in CI users with excellent and poor cortical responses, the FFR response amplitude in speech-ABR differed between the two groups, and other peaks did not show any significant difference. Speech stimulus has higher energy in the periodic region, and higher energy represents better neural processing (19). It has been found that insufficient neural excitation caused by the disorder in phase-locking leads to the decreased amplitude of responses.

Spectral-domain analysis was also performed in this study. This analysis shows to what extent the responses fall within each given frequency band over a range of frequencies in CI users (18). Spectral analysis was performed through the fast Fourier transform (FFT) algorithm decomposing a complex wave into sine waves (20). Spectral analysis of response measured the amplitude and precision of the phase of locking of F0, F1, and HF in the first formant of speech stimuli. Analyses indicated that total energy occurring around F0, F1, and HF was lower in the CI-poor group than in the CI-good group. Prior studies have indicated that encoding of F0 has an essential role in identifying the speaker and emotional tone of voice. This component has a low-frequency structure of speech, results from the periodic beating of sound, and is finally indicated as the perceived pitch of the subject voice.

Furthermore, F1 and HF have an important role in the perception of the vowel of speech sound and also provide phonetic information about speech sound (21). In the current study, it was demonstrated that processing of fundamental frequency was changed in CI users with poor speech recognition in noise performance compared to CI users with good recognition; thus, this result provided the evidence that fundamental frequency encoding is tracked by speech in noise in CI users. Also, the results of this study showed that a lower subcortical representation of F0, F1, and HF in spectral-domain in CI users could be physiological evidence for weaker encoding of the signal pitch.

Additionally, recognition of words was measured in quiet and noise conditions. As expected, recognition of the words in quiet was easier than noise so that there were no significant differences between both groups of CI users (p<0.005). This similarity is justified by several factors. It was observed that the top-down effect used in CI users helped them to predicate the words. This hypothesis was previously suggested by Ahissar (2007), who stated that language knowledge allows listeners to make predictions about the structure, and this phenomenon would be applicable to perception in a quiet condition similar to noise condition, especially if the signal is degraded due to processing limitations (22). Given this, the relationship between subcortical and cortical is reciprocal; cognitive-based processes, like attention, language experience, and memory likely influence the subcortical structure through corticofugal enhancement, and on the other hand, a deficit in subcortical processing results in a degraded input signal to the auditory cortex and causes the corticofugal function to be powerless. CI users with poor speech recognition in noise performance likely have a deficiency in the encoding of speech sound, leading to a weak relationship between cortical and subcortical structures which finally results in poor performance in a speech in noise ability (21, 23, 24). However, when background noise is added, the top-down effect decreases; thus, in this situation, the use of acoustical features will be essential for speech recognition in noise performance. Previous studies have shown that speech in noise perception is related to brainstem encoding of speech stimuli. It has been reported that among different processing at the brainstem level, encoding of fundamental frequency plays an important role in speech recognition in noise performance (25, 26). Therefore, considering these results, the difference observed in speech recognition in noise performance between two groups of CI users in this study may be attributable to at least in part different processing of fundamental frequency of speech stimuli at brainstem level (27). 

## In conclusion

 the results of the study revealed that CI users who showed poor auditory performance, especially in noise, had deficits in the encoding of the periodic portion of speech signals at the brainstem level. Also, pitch processing, including F0, H1, and HF was weaker in CI users with poor speech recognition in noise performance than those with good performance. Auditory evoked potentials, such as speech-ABR test as objective, reliable, and fast method would be useful for determining the CI users who show abnormality in speech processing at the brainstem level. Administrating auditory training based on speech in noise program and monitoring by the speech-ABR test is recommended for CI users in future research, especially those with poor speech recognition in noise performance.

## Author’s Contribution

Proposed the main concept and idea of the research, performed the research, and wrote the paper. Contributed to the study design, data collection, and interpretation of the results. contributed to statistical analysis, also contributed to data collection, statistical analysis, and interpretation of the results. All authors agreed to be accountable for all aspects of the work, ensuring that questions related to the accuracy or integrity of any part of the work were appropriately investigated and resolved.

## Conflict of Interest

The authors declared that there are no conflicts of interests
